# Sex and Age Differences in Patient-Reported Acute Stroke Symptoms

**DOI:** 10.3389/fneur.2022.846690

**Published:** 2022-03-21

**Authors:** Heidi S. Eddelien, Jawad H. Butt, Thomas Christensen, Anne K. Danielsen, Christina Kruuse

**Affiliations:** ^1^Department of Neurology, Copenhagen University Hospital - Herlev and Gentofte, Copenhagen, Denmark; ^2^Department of Clinical Medicine, University of Copenhagen, Copenhagen, Denmark; ^3^Department of Cardiology, Copenhagen University Hospital - Rigshospitalet, Copenhagen, Denmark; ^4^Department of Neurology, Nordsjællands Hospital - Rigshospitalet, Copenhagen, Denmark; ^5^Department of Gastroenterology, Copenhagen University Hospital - Herlev and Gentofte, Copenhagen, Denmark

**Keywords:** stroke, signs and symptoms, sex diferences, prehospital delay time, behavior

## Abstract

**Background:**

Identification of sex- and age-related differences in the presentation of atypical symptoms at stroke onset may reduce prehospital delay and improve stroke treatment if acknowledged at first contact.

**Aim:**

To explore sex- and age-related differences in patient-reported typical and atypical symptoms of a stroke.

**Methods:**

We used data from a cross-sectional survey at two non-comprehensive stroke units in the Capital Region of Denmark. Patient-reported symptoms, stroke knowledge, and behavioral response were analyzed by the Chi-square test or a Fisher's exact test separated by sex. Multivariable logistic regression adjusted for covariates were used to explore sex- and age-related differences according to each patient-reported typical or atypical symptoms.

**Results:**

In total, 479 patients with acute stroke were included (median age 74 years [25th to 75th percentile: 64–80], and 40.1% were women). Female sex was associated with higher odds of presenting with atypical symptoms, such as loss of consciousness (OR 2.12 [95% CI 1.08–4.18]) and nausea/vomiting (OR 2.33 [95% CI 1.24–4.37]), and lower odds of presenting with lower extremity paresis (OR 0.59 [95% CI 0.39–0.89). With each year of age, the odds decreased of presenting with sensory changes (OR 0.95 [95% CI 0.94–0.97]) and upper extremity paresis (OR 0.98 [95% CI 0.96–0.99]), whereas odds of presenting with dysphagia (OR 1.06 [95% CI 1.02–1.11]) increased.

**Conclusions:**

Patients of female sex and younger age reported on admission more frequently atypical stroke symptoms. Attention should be drawn to this possible atypical first presentation to facilitate correct identification and early stroke revascularization treatment to improve the outcome for both sexes.

## Introduction

Identification of sex- and age-related differences in the presentation of atypical symptoms at stroke onset may improve stroke treatment if acknowledged at first contact and reduce prehospital delay. Failed recognition of stroke upon presentation may cause delayed treatment with reduced clinical outcome (1–4). Currently, a small but increasing proportion of all ischemic stroke patients are treated with revascularization therapy, a well-established treatment worldwide (5–7). A frequent cause of failure to receive revascularization therapy within the required time window is a prehospital delay, either patient or system related. Patient delay is often associated with failure in symptom recognition or reluctance to respond acutely to symptoms. In system delay, much has improved in door-to-needle time at comprehensive stroke units but is still largely affected by missed symptom recognition by the health professionals at first contact (8–11). Stroke symptoms can be categorized as typical (12) (e.g., hemiparesis, facial palsy, visual, or language disturbances) or atypical (e.g., headache, dizziness, confusion, or sensory symptoms) (13), where the latter induce a significant risk of missing the stroke diagnosis.

Minimizing response time and implementing fast-track treatment of stroke is the key to reducing the impact of stroke on death and disability worldwide (14). Previous studies suggest that patients of female sex present a different profile of stroke symptoms on admission compared to the male sex. However, these studies focused on symptoms identified by health professionals and not those reported by the patients or bystanders. Symptoms recognized by health professionals may be different from those experienced and reported by the patient and bystanders potentially unaware of stroke-related symptoms (15–19). Knowledge is scarce on which acute stroke symptoms are reported by the patient or the bystander on admission. In this study, we have focused on the sex- and age-related differences in patient-reported symptoms which cause them to react and present to the prehospital health system. Older patients (>80 years of age) seem to present more frequently with typical symptoms, such as aphasia and hemiplegia (18). Headache and nausea are reported more commonly in younger patients, even after controlling for concomitant migraine (19). It remains to be seen if increased age amplifies this difference in the patient-reported symptoms (9). We hypothesized that patient-reported atypical symptoms at the onset of stroke were more frequent in the female sex and that the distribution of typical and atypical stroke symptoms varied with increasing age. Accordingly, we aimed to explore the sex- and age-related differences in patient-reported symptoms of a stroke.

## Methods

### Study Design and Setting

This study is a *post-hoc* analysis of data from a cross-sectional survey performed at two non-comprehensive stroke units in the Capital Region of Denmark, Herlev Gentofte Hospital, and Nordsjællands Hospital. Study design and methods have previously been published (11).

### Study Population

Patients with symptoms of acute stroke or transient ischemic attack (TIA) were enrolled immediately after admission to the stroke unit, albeit before completion of a full diagnostic workup for stroke. All diagnoses [International Classification of Diseases codes: I61: non-traumatic intracerebral hemorrhage (ICH); I63: ischemic stroke (IS); or G45. TIA] were confirmed by a neurologist supported by neuroimaging (CT and MRI scans). Enrolled patients fulfilled the following inclusion criteria: (1) admitted directly to a non-comprehensive stroke unit or transferred from a comprehensive or primary stroke center after revascularization therapy, (2) age ≥ 18 years, and (3) obtained written consent from the patient. Exclusion criteria were patients with (1) a subarachnoid hemorrhage, (2) an in-hospital stroke, (3) a non-stroke diagnosis, or (4) symptom onsets abroad. Only the first event was included in case of recurrent stroke during the inclusion period.

### Data Collection

Data were collected from February 2018 to June 2018 and September 2018 to January 2019 at Herlev Gentofte Hospital and Nordsjællands Hospital, both located in The Capital Region of Denmark. Medical records and emergency medical service (EMS) data supported patients' responses. Data were managed in a Research Electronic Data Capture, REDCAP, system (REDCap consortium, Vanderbilt University, United States of America, v9.1.0 hosted by The Capital Region of Denmark) (20).

### Variables

Typical patient-reported symptoms were defined according to the American Stroke Association's stroke warning signs and symptoms “BEFAST save a life,” covering Balance, Eyes, Face, Arm, Speech (and Time). BEFAST describes factors associated with the need to call EMS immediately for treatment evaluation (12). Atypical stroke symptoms were defined as symptoms not included in BEFAST (e.g., pain, loss of consciousness, unclassifiable neurological symptoms, and non-neurological symptoms) (13, 21). Patient-reported symptoms were categorized by the interviewer into predefined medical terms.

### Statistical Methods

#### Analysis

Baseline characteristics were summarized as frequencies with percentages or medians with interquartile ranges (IQR), and differences were tested using the Chi-square test or Fisher's exact test for categorical variables and the Wilcoxon test for continuous variables. To explore sex- and age-related differences in patient-reported typical and atypical stroke symptoms, multivariable logistic regression models were used to estimate odds ratios (OR) with 95% confidence intervals (CI) for each symptom. Models were adjusted for stoke severity assessed by the Scandinavian Stroke Scale (severe 0–25 points, moderate 26–42 points, and mild 43–58 points) (22), stroke localization (right hemisphere, left hemisphere, or bilateral, brainstem, cerebellum), a history of hypertension, diabetes (yes/no), atrial fibrillation (yes/no), and hypercholesterolemia (yes/no). Age was included as a continuous variable. Male sex was the reference group in all statistic models. All statistical analyses were performed using SAS statistical software version 9.4 (SAS Institute, Cary, NC, United States). A two-sided significance level was set at alpha < 0.05. There were no missing data on stroke symptoms.

#### Sensitivity Analysis

To test the robustness of our findings, we conducted several sensitivity analyses. In the first analysis, all symptoms were grouped into two categories: typical and atypical symptoms and examined for differences in age and sex. In the second analysis, age was included as a categorical variable with the following age groups 18–59 years, 60–74 years, and 75+. In the third analysis, age was included in 5-year intervals.

### Ethics

The study was approved by the Capital Region's Ethics Committee (no. 2012-58-004) and the Danish Data Protection Agency (no. 2012-58-0004; internal reference: HGH-2017-110, I-Suite no. 06014). Patients provided written informed consent before interviews.

## Results

### Patient Characteristics

**Figure 1:**
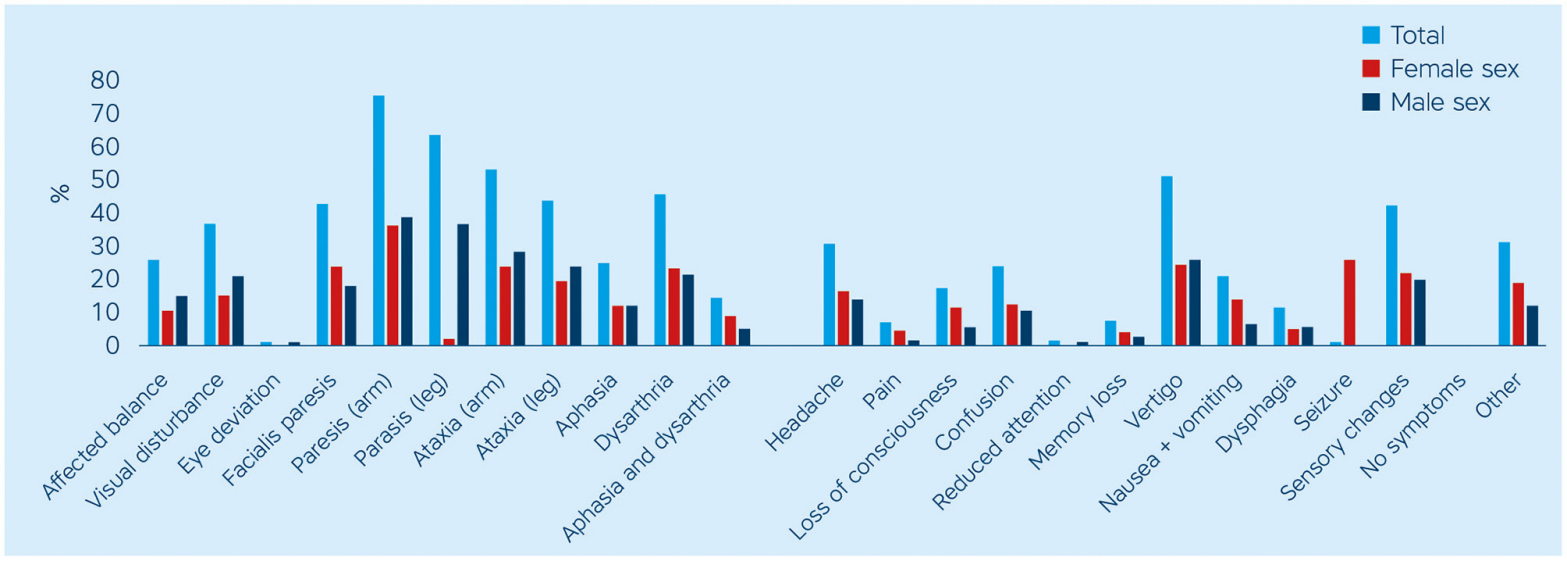
Acute patient-reported typical and atypical stroke symptoms, %.

The process of enrollment is described in detail elsewhere (11). In total, 479 patients with stroke or TIA were included (40.1% female), and the median age was 74 years (64–80) with no significant age-related difference between sexes. Baseline characteristics are summarized in Table 1, and Table 2 summarizes patient-reported symptoms, stroke knowledge, behavioral response, arrival time, and treatment. The proportions of acute stroke symptoms are displayed in Figure 1. There were no differences between women and men with respect to stroke knowledge, behavioral response, hospital arrival within 180 min from symptom onset, or stroke treatment.

**Table 1:** Patient characteristics.

**Variables**	**Women** ***N* = 192** **(40.10%)**	**Men** ***N* = 287** **(59.90%)**	***P*-value**
**Demographics**			
-Age, median 25th−75th percentile	75 (65–82)	73 (64–79)	0.25
**Level of education**			
-Basic	78 (40.63)	112 (39.02)	<0.001
-Further	95 (49.48)	110 (38.33)	
-Higher	18 (9.38)	65 (22.65)	
-Not available	1 (0.52)	0 (0.00)	
**Living arrangements**			
-Living with someone	92 (47.92)	198 (68.99)	<0.001
-Living alone	100 (52.08)	89 (31.01)	
**Scandinavian stroke scale score^a^**			
-Mild	159 (82.81)	247 (86.06)	0.60
-Moderate	24 (12.50)	28 (9.76)	
-Severe	9 (4.69)	12 (4.18)	
Type of stroke			
-I61: Nontraumatic intracerebral hemorrhage	16 (8.33)	23 (8.01)	0.12
-I63: Cerebral infarction	114 (59.38)	195 (67.94)	
-G45: Transient cerebral ischemic attacks	62 (32.29)	69 (24.04)	
**Stroke location**			
-Center hemisphere	81 (42.19)	107 (37.28)	0.48
-Right hemisphere	66 (34.38)	101 (35.19)	
-Bilateral, brainstem, cerebellum	45 (23.44)	79 (27.53)	
**Risk factors, history of**			
-Hypertension	110 (57.29)	154 (53.66)	0.43
-Diabetes	14 (7.29)	40 (13.94)	0.02
-Atrial fibrillation	40 (20.83)	63 (21.95)	0.77
-Hypercholesterolemia	89 (46.35)	128 (44.60)	0.71
-Acute myocardial infarct	9 (4.69)	27 (9.41)	0.05
-Claudication	19 (9.90)	21 (7.32)	0.32
-Carotid stenosis	18 (9.38)	18 (6.27)	0.21
-Heart failure	7 (3.65)	30 (10.45)	0.01
-Sleep apnea	7 (3.65)	29 (10.10)	0.01
-Prior Stroke	35 (18.23)	59 (20.56)	0.53
-Smoking			
- Current	40 (20.83)	67 (23.34)	0.04
- Former	72 (37.50)	133 (46.34)	
- Never	80 (41.67)	87 (30.31)	
**Pre-hospital medication for stroke comorbidity**			
-≥1	126 (65.63)	178 (62.02)	0.42
-None	66 (34.38)	109 (37.98)	

a *Scandinavian Stroke Scale classified as severe (0–25 points), moderate (26–42 points), and mild (43–58 points)*.

**Table 2:** Patient-reported symptoms, stroke knowledge, behavioral response, arrival time, and treatment.

**Variables**	**Women** ***N* = 192 (40.10%)**	**Men** ***N* = 287 (59.90%)**	***P*-value**
**Typical stroke symptom**			
-Affected balance	21 (10.94)	44 (15.33)	0.17
-Visual disturbance	30 (15.63)	61 (21.25)	0.12
-Eye deviation	0 (0.00)	4 (1.39)	0.10
-Facial paresis	47 (24.48)	53 (18.47)	0.11
-Paresis (arm)	70 (36.46)	112 (39.02)	0.57
-Paresis (leg)	51 (26.56)	106 (36.93)	0.02
-Ataxia (arm)	47 (24.48)	83 (28.92)	0.28
-Ataxia (leg)	38 (19.79)	69 (24.04)	0.27
-Aphasia	24 (12.50)	36 (12.54)	0.99
-Dysarthria	46 (23.96)	63 (21.95)	0.61
-Aphasia and dysarthria	18 (9.38)	16 (5.57)	0.11
**Atypical stroke symptoms**			
-Headache	32 (16.67)	42 (14.63)	0.55
-Pain	10 (5.21)	6 (2.09)	0.06
-Loss of consciousness	23 (11.98)	17 (5.92)	0.02
-Confusion	25 (13.02)	32 (11.15)	0.54
-Reduced attention	1 (0.52)	4 (1.39)	0.36
-Memory loss	9 (4.69)	9 (3.14)	0.38
-Vertigo	48 (25.00)	75(26.13)	0.78
-Nausea + vomiting	28 (14.58)	20 (6.97)	0.01
-Dysphagia	11 (5.73)	18 (6.27)	0.81
-Seizure	2 (1.04)	1 (0.35)	0.35
-Sensory changes	43 (22.40)	58 (20.21)	0.57
-No symptoms	0 (0.00)	1 (0.35)	0.41
-Symptoms that did not fit in the predefined symptom boxes	37 (19.27)	36 (12.54)	0.04
**Typical acute stroke symptoms FAST^a^**			
-≥1	95 (49.48)	142 (49.48)	0.10
-None	97 (50.52)	145 (50.52)	
**Typical acute stroke symptoms BEFAST^b^**			
-≥1	97 (50.52)	145 (50.52)	0.10
-None	95 (49.48)	142 (49.48)	
**Perceived severity of symptoms^c^**			
- < median 25	81 (42.19)	154 (53.66)	0.05
-≥ median 25	100 (52.08)	120 (41.81)	
-Not available	11 (5.73)	13 (4.53)	
**Stroke recognition**			
-Yes	162 (84.38)	251 (87.46)	0.61
-No	28 (14.58)	33 (11.50)	
-Not available	2 (1.04)	3 (1.05)	
**Prior knowledge of acute stroke therapy**			
-Yes	108 (56.25)	158 (55.05)	0.79
-No	84 (43.75)	129 (44.95)	
**Help seeking behavior, first contact**			
-Emergency medical service	66 (34.38)	81 (28.22)	0.28
-OOH-PC	43 (22.40)	78 (27.18)	
-General practitioner	54 (28.13)	86 (29.97)	
-Home Care	6 (3.13)	5 (1.74)	
-Out-patient Clinic	9 (4.69)	7 (2.44)	
-Family, friend, neighbor, co-workers	12 (6.25)	27 (9.41)	
-Unknown bystander	1 (0.52)	0 (0.00)	
-None	1 (0.52)	3 (1.05)	
**Arrival time**			
-Within 180 min from onset	70 (36.46)	104 (36.24)	0.96
**Treatment**			
-Thrombolysis	26 (13.54)	31 (10.80)	0.36
-Thrombectomy	5 (2.60)	7 (2.44)	0.91

a *FAST acronym for Face, Arm, Speech, Time*.

b *BEFAST acronym for Balance, Eye, Face, Arm, Speech, Time*.

c *Patient-perceived symptom severity was rated on a scale from 0 to 100, where 100 was most severe*.

### Primary Outcome

In multivariable logistic regression analyses, female sex, compared with male sex, was associated with higher odds of presenting with loss of consciousness [adjusted OR 2.12 (95% CI 1.08–4.18)] and nausea/vomiting [adjusted OR 2.33 (95% CI 1.24–4.37)], but lower odds of presenting with lower extremity paresis [adjusted OR 0.59 (95% CI 0.39–0.89)] (Table 3). With increase in age each year, the odds were lower of presenting with sensory changes [adjusted, OR 0.95 (95% CI 0.94–0.97)] and upper extremity paresis [adjusted OR 0.98 (95% CI 0.96–0.99)]. The odds of presenting with dysphagia [adjusted OR 1.06 (95% CI 1.02–1.11)] increased with each year of age (Table 4).

**Table 3:** Sex-related differences in patient-reported stroke symptoms (continuous).

	**OR**	**95 % confidence intervals**
**Typical stroke symptoms (BEFAST)**		
Affected balance	0.69	0.39–1.21
Vision disturbance	0.65	0.39–1.09
Facials paresis	1.45	0.91–2.31
Paresis, (arm)	0.88	0.59–1.31
Paresis, (leg)	0.59	0.39–0.89
Ataxia, (arm)	0.81	0.53–1.23
Ataxia, (leg)	0.76	0.48–1.20
Aphasia	0.98	0.55–1.75
Dysarthria	1.05	0.67–1.65
Aphasia + Dysarthria	1.86	0.88–3.91
**Atypical stroke symptoms**		
Headache	1.18	0.71–1.98
Pain	2.76	0.96–7.92
Loss of consciousness	2.12	1.08–4.18
Confusion	1.21	0.68–2.14
Reduced attention	0.32	0.03–3.17
Memory loss	1.63	0.62–4.32
Vertigo	0.99	0.64–1.53
Nausea + vomiting	2.33	1.24–4.37
Dysphagia	0.82	0.36–1.86
Seizure	3.85	0.31–47.56
Sensory changes	1.20	0.76–1.92
Other	1.76	1.06–2.95

*Reference category men. Adjusted for age, stroke severity, stroke localization, history of hypertension, diabetes, atrial fibrillation, and hypercholesterolemia. Eye deviation and no symptoms were removed from the analysis, due to zero observation in one of the groups*.

**Table 4:** Age-related differences in patient-reported stroke symptoms with each year increase.

	**OR**	**95 % confidence intervals**
**Typical stroke symptoms (BEFAST)**		
Affected balance	1.01	0.98–1.03
Vision disturbance	0.98	0.96–1.00
Facials paresis	1.00	0.98–1.02
Paresis, (arm)	0.98	0.96–1.00
Paresis, (leg)	1.00	0.99–1.02
Ataxia, (arm)	0.99	0.97.1.01
Ataxia, (leg)	1.00	0.98–1.02
Aphasia	1.01	0.99–1.04
Dysarthria	1.01	0.99–1.03
Aphasia + Dysarthria	1.02	0.98–1.05
**Atypical stroke symptoms**		
Headache	0.98	0.96–1.00
Pain	0.99	0.95–1.03
Loss of consciousness	1.00	0.97–1.03
Confusion	1.02	0.99–1.05
Reduced attention	0.98	0.90–1.07
Memory loss	1.01	0.97–1.06
Vertigo	0.99	0.98–1.01
Nausea + vomiting	0.99	0.96–1.02
Dysphagia	1.06	1.02–1.11
Seizure	1.04	0.93–1.16
Sensory changes	0.95	0.94–0.97
Other	0.98	0.96–1.01

*Reference category men. Adjusted for sex, stroke severity, stroke localization, history of hypertension, diabetes, atrial fibrillation, and hypercholesterolemia. Eye deviation and no symptoms were removed from the analysis, due to zero observation in one of the groups*.

### Sensitivity Analyses

When comparing typical vs. atypical symptoms, the male sex was not significantly associated with higher odds of presenting with either typical or atypical symptoms [adjusted OR 1.55, (95% CI 0.84–2.87)]. When age was included as a categorical variable or included in 5-year intervals (Supplementary Tables I, IIII online-only data supplement) our primary findings were confirmed. Including age as a categorical variable, the odds for presenting with sensory changes (atypical symptom) were lower when comparing the age group 18–59 years with 60–74 years [adjusted OR 0.52 (95% CI 0.30–0.92)], (Supplementary Table II online-only data supplement). When we compared the age group 18–59 years with 75+ years, the results from the primary analysis were confirmed (Supplementary Table III online-only data supplement). Including age in 5-year intervals, the odds of presenting with upper extremity paresis (typical symptom) and sensory changes (atypical symptom) were significantly lower with each 5-years increase in age [adjusted, OR 0.89 (95% CI 0.82–0.97)] and [adjusted, OR 0.79 (95% CI 0.72–0.87)] (Supplementary Table V online-only data supplement). We performed the sub-analysis according to the type of stroke by sex. In population I61, female sex was significantly associated with loss of consciousness (atypical; Supplementary Table VI online-only data supplement). In population I63, female sex was significantly associated with nausea + vomiting (atypical; Supplementary Table VII online-only data supplement). In population G45, male sex was significantly associated with affected balance (typical; Supplementary Table VIII online-only data supplement).

## Discussion

In this cross-sectional two-center survey, we explored sex- and age-related differences in patient-reported typical and atypical stroke symptoms in a stroke and TIA population. Our study yielded the following finding: Female sex was associated with higher odds of reporting atypical stroke symptoms, such as loss of consciousness and nausea/vomiting, and lower odds of reporting typical stroke symptoms, such as lower extremity paresis compared to the male sex, which confirmed our hypothesis. With increasing age, we found that sensory changes (atypical symptom) and upper extremity paresis (typical symptom) were less frequently reported, whereas dysphagia (atypical symptom) was more frequent with increasing age, which largely confirmed our hypothesis.

The chain of survival and the acute treatment of stroke and TIA have important similarities with that of acute coronary syndrome. To reduce morbidity and mortality, correct interpretation and action on acute symptoms are essential to ensure timely and correct revascularization therapy. In symptomatic manifestations of acute coronary syndrome, female sex is associated with frequent atypical presentations of symptoms, for example, unusual fatigue, dyspnea, neck and throat pain, and pain between the shoulder blades. This diversity of acute symptoms led to delay in identification and interpretation (23, 24). To which extent this diversity in symptom presentation may apply to stroke need to be addressed. In our study, female sex was associated with the initial presentation of atypical symptoms, such as loss of consciousness and nausea/vomiting at stroke onset. The findings on patient-reported symptoms were in line with studies where acute symptoms were identified by health professionals, hence female sex is associated with symptoms, such as loss of consciousness (17, 25, 26) and nausea/vomiting (27). Studies further reported that female sex, in comparison to the male sex, tended to present with other atypical symptoms identified by health professionals, such as dysphagia (25, 26, 28), headache (15, 16), mental status change (21, 29), and seizure (27). The findings that the female sex entailed a variety of atypical symptoms upon onset of stroke highlight the risk of misinterpretation symptoms as non–stroke related. In one study, female sex was associated with a longer hospital arrival time, including a decreased likelihood of reperfusion therapy, primarily due to patient-dependent delay (30). However, when adjusted for age, stroke severity, and co-habitant status, the sex difference in prehospital delay disappeared (30). In our univariate analysis 36% of both women and men arrived within 180 min from the onset. We could not confirm a significant sex difference in timely hospital arrival within 180 min. Some atypical presentations may be interpreted as severe, such as loss of consciousness or seizure, which caused rapid contact to emergency services. This may contribute to why sex differences in symptoms presentation did not significantly affect arrival times in the group. Having a bystander or co-habiting at the onset of stroke was previously associated with an increased chance of stroke recognition and immediate contact to emergency medical services (31, 32). In our study, the proportion of female sex living alone at stroke onset was significantly higher compared to the male sex, but this did not affect arrival times. Nonetheless, perception of symptom severity was associated with timely hospital arrival in other studies (32, 33).

The American Heart Association warning signs for myocardial infarction included a cautionary statement in their campaign, stressing the fact that the female sex is more likely to experience atypical symptoms (34). Previous stroke awareness campaigns focused on improving knowledge of typical symptoms and increasing behavioral response, but the effect on timely arrival has so far been inconclusive (35, 36). Future stroke awareness campaigns should be tailored to address that female sex may associate also with atypical presentations of stroke, to embrace sex differences in stroke care (37). We could not confirm any sex differences in stroke recognition, prior knowledge of stroke therapy, or help-seeking behavior. This could be due to no launch of a focused stroke awareness campaign before our study, and the number of included patients was relatively small.

Stroke patients above the age of 75 years presented more frequently with dysphagia (atypical symptom) when identified by health professionals (28). This was aligned when dysphagia was patient-reported in our study. Other atypical symptoms, such as coma, aphasia, and cerebellar dysfunction, have also been associated with stroke patients above 80 years (18). These findings were not confirmed in this study. Interestingly, headache, nausea/vomiting, and sensory deficits (atypical symptoms) appeared to be more common in the female sex between 18 and 44 years, even after controlling for a diagnosis of migraine and for age (19). Results on stroke patients younger than 55 years showed that almost 25% of strokes were not identified when clustering acute stroke symptoms according to typical symptoms (19). Furthermore, it should be stressed that the outcomes of atypically presenting stroke patients are not necessarily benign, if the symptoms are misdiagnosed (38).

### Limitations

We aimed to reduce recall bias by including patients as early as possible after stroke onset. A selection bias cannot be dismissed as there was an overrepresentation of patients with mild stroke, either due to early patient discharge, transfer to other departments if there was a change in clinical condition before inclusion was possible which could omit mild strokes or severely affected patients. The included population represented a broad stroke population arriving at non-comprehensive stroke units. The hypothesis of this study was devised after the overall study was designed but before the end of this study. Analyses were therefore exploratory and mainly applicable as hypothesis-generating.

## Conclusions

The current study confirmed our hypothesis that sex differences in patient reporting of acute stroke symptoms exist. These findings were in accordance with previous studies investigating physician-reported symptoms, where the female sex presented with atypical symptoms, such as loss of consciousness and nausea/vomiting. Recognition of stroke and correct response to symptoms pose a particular challenge in early treatment when atypical stroke symptoms are reported. It needs to be further assessed to which extent sex- and age-related differences in symptoms of acute stroke influence interpretation of symptoms, behavioral motivators, and barriers for first contact to emergency medical service.

## Data Availability Statement

The raw data supporting the conclusions of this article will be made available by the authors, without undue reservation.

## Ethics Statement

The studies involving human participants were reviewed and approved by Capital Region's Ethics Committee, Danish Data Protection Agency. The patients/participants provided their written informed consent to participate in this study.

## Author Contributions

CK and AD were responsible for protocol development, gathering ethical approval, and data permission. Sub study on sex and age was conceived by HE and CK. HE and TC (only Nordsjællands Hospital) were involved in patient recruitment and data collection. HE, JB, and CK in data analysis. HE wrote the first draft of the manuscript. All authors made critical revisions to the manuscript and approved its final version.

## Funding

This work was supported by TrygFonden, application ID 128669 and by the Novo Nordisk Foundation Borregaard stipend, grant number NNF18OC0031840.

## Conflict of Interest

The authors declare that the research was conducted in the absence of any commercial or financial relationships that could be construed as a potential conflict of interest.

## Publisher's Note

All claims expressed in this article are solely those of the authors and do not necessarily represent those of their affiliated organizations, or those of the publisher, the editors and the reviewers. Any product that may be evaluated in this article, or claim that may be made by its manufacturer, is not guaranteed or endorsed by the publisher.
