# Supplementary material for: Sex and Age Differences in Patient-Reported Acute Stroke Symptoms
Source: Front Neurol. 2022 Mar 21;13:846690. doi: 10.3389/fneur.2022.846690 (PMC8978710; doi:10.3389/fneur.2022.846690)
Supplement: Supplementary file 1 [file Table_1.DOCX]

**SUPPLEMENTRY MATERIAL**

**Data supplement Table I Sex differences in patient-reported stroke symptoms (categorically)**

| **Typical stroke symptoms (BEFAST)** | **OR** | **95 % confidence intervals** |
| --- | --- | --- |
| Affected balance | 0.68 | 0.39-1.21 |
| Vision disturbance | 0.65 | 0.39-1.08 |
| Facials paresis | 1.44 | 0.90-2.29 |
| Paresis, (arm) | 0.89 | 0.60-1.34 |
| Paresis, (leg) | 0.59 | 0.39-0.90 |
| Ataxia, (arm) | 0.82 | 0.54-1.25 |
| Ataxia, (leg) | 0.75 | 0.47-1.18 |
| Aphasia | 0.97 | 0.54-1.74 |
| Dysarthria | 1.03 | 0.66-1.62 |
| Aphasia + Dysarthria | 1.78 | 0.84-3.78 |

*reference category men. Adjusted for sex, stroke severity, stroke localization, history of hypertension, diabetes, atrial fibrillation, and hypercholesterolemia. Eye deviation and no symptoms were removed from analysis, due to zero observation in one of the groups.

**(categories years, 1= 18-59, 2= 60-74, 3= 75+)**

| **Atypical stroke symptoms** | **OR** | **95 % confidence intervals** |
| --- | --- | --- |
| Headache | 1.18 | 0.70-1.97 |
| Pain | 2.72 | 0.94-7.89 |
| Loss of consciousness | 2.10 | 1.06-4.15 |
| Confusion | 1.17 | 0.66-2.09 |
| Reduced attention | 0.32 | 0.03-3.26 |
| Memory loss | 1.60 | 0.60-4.26 |
| Vertigo | 1.01 | 0.65-1.57 |
| Nausea + vomiting | 2.30 | 1.23-4.32 |
| Dysphagia | 0.84 | 0.37-1.89 |
| Seizure | 3.96 | 0.31-50.71 |
| Sensory changes | 1.23 | 0.77-1.96 |
| Other | 1.76 | 1.06-2.95 |

*reference category men. Adjusted for sex, stroke severity, stroke localization, history of hypertension, diabetes, atrial fibrillation, and hypercholesterolemia. Eye deviation and no symptoms were removed from analysis, due to zero observation in one of the groups.

**(categories years, 1= 18-59, 2= 60-74, 3= 75+)**

**Data supplement Table II Age differences in patient-reported stroke symptoms** **(categorically) 60 - 74 years vs. 18 - 59 years**

| **Typical stroke symptoms (BEFAST)** | **OR** | **95 % confidence intervals** |
| --- | --- | --- |
| Affected balance | 1.03 | 0.48-2.19 |
| Vision disturbance | 0.60 | 0.32-1.14 |
| Facials paresis | 0.72 | 0.38-1.34 |
| Paresis, (arm) | 0.84 | 0.50-1.43 |
| Paresis, (leg) | 1.14 | 0.65-2.00 |
| Ataxia, (arm) | 0.90 | 0.52-1.56 |
| Ataxia, (leg) | 0.62 | 0.34-1.12 |
| Aphasia | 0.82 | 0.37-1.84 |
| Dysarthria | 0.74 | 0.40-1.37 |
| Aphasia + Dysarthria | 0.86 | 0.28-2.71 |

*reference category men. Adjusted for sex, stroke severity, stroke localization, history of hypertension, diabetes, atrial fibrillation, and hypercholesterolemia. Eye deviation and no symptoms were removed from analysis, due to zero observation in one of the groups.

**(categorically, 1= 18-59, 2= 60-74, 3= 75+)**

| **Atypical stroke symptoms** | **OR** | **95 % confidence intervals** |
| --- | --- | --- |
| Headache | 0.72 | 0.37-1.40 |
| Pain | 1.17 | 0.28-4.97 |
| Loss of consciousness | 0.69 | 0.28-1.73 |
| Confusion | 0.81 | 0.34-1.94 |
| Reduced attention | 0.45 | 0.06-3.51 |
| Memory loss | 1.34 | 0.26-7.00 |
| Vertigo | 1.23 | 0.68-2.20 |
| Nausea + vomiting | 0.82 | 0.35-1.94 |
| Dysphagia | 1.67 | 0.33-8.33 |
| Seizure | ˃999.10 | ˂0.00- ˃999.10 |
| Sensory changes | 0.52 | 0.30-0.92 |
| Other | 0.88 | 0.45-1.71 |

*reference category men. Adjusted for sex, stroke severity, stroke localization, history of hypertension, diabetes, atrial fibrillation, and hypercholesterolemia. Eye deviation and no symptoms were removed from analysis, due to zero observation in one of the groups.

**(categorically, 1= 18-59, 2= 60-74, 3= 75+)**

**Data supplement Table III Age differences in patient-reported stroke symptoms** **(categorically) 75+ years vs. 18 – 59 years**

| **Typical stroke symptoms (BEFAST)** | **OR** | **95 % confidence intervals** |
| --- | --- | --- |
| Affected balance | 1.13 | 0.53-2.39 |
| Vision disturbance | 0.54 | 0.28-1.04 |
| Facials paresis | 0.76 | 0.41-1.42 |
| Paresis, (arm) | 0.48 | 0.28-0.83 |
| Paresis, (leg) | 1.02 | 0.58-1.78 |
| Ataxia, (arm) | 0.63 | 0.36-1.11 |
| Ataxia, (leg) | 0.68 | 0.38-1.23 |
| Aphasia | 1.08 | 0.49-2.37 |
| Dysarthria | 1.01 | 0.55-1.85 |
| Aphasia + Dysarthria | 1.60 | 0.54-4.72 |

*reference category men. Adjusted for sex, stroke severity, stroke localization, history of hypertension, diabetes, atrial fibrillation, and hypercholesterolemia. Eye deviation and no symptoms were removed from analysis, due to zero observation in one of the groups.

**(categorically, 1= 18-59, 2= 60-74, 3= 75+)**

| **Atypical stroke symptoms** | **OR** | **95 % confidence intervals** |
| --- | --- | --- |
| Headache | 0.60 | 0.30-1.18 |
| Pain | 1.27 | 0.30-5.32 |
| Loss of consciousness | 0.76 | 0.31-1.87 |
| Confusion | 1.61 | 0.72-3.59 |
| Reduced attention | 0.24 | 0.02-3.41 |
| Memory loss | 2.44 | 0.51-11.66 |
| Vertigo | 0.82 | 0.45-1.50 |
| Nausea + vomiting | 0.80 | 0.34-1.88 |
| Dysphagia | 5.10 | 1.14-22.89 |
| Seizure | ˃999.10 | ˂0.00- ˃999.10 |
| Sensory changes | 0.23 | 0.13-0.43 |
| Other | 0.68 | 0.34-1.36 |

*reference category men. Adjusted for sex, stroke severity, stroke localization, history of hypertension, diabetes, atrial fibrillation, and hypercholesterolemia. Eye deviation and no symptoms were removed from analysis, due to zero observation in one of the groups.

**(categorically, 1= 18-59, 2= 60-74, 3= 75+)**

**Data supplement Table IIII Sex differences in patient-reported stroke symptoms (5 years interval)**

| **Typical stroke symptoms (BEFAST)** | **OR** | **95 % confidence intervals** |
| --- | --- | --- |
| Affected balance | 0.69 | 0.39-1.21 |
| Vision disturbance | 0.65 | 0.39-1.09 |
| Facials paresis | 1.45 | 0.91-2.31 |
| Paresis, (arm) | 0.88 | 0.59-1.31 |
| Paresis, (leg) | 0.59 | 0.39-0.89 |
| Ataxia, (arm) | 0.81 | 0.53-1.23 |
| Ataxia, (leg) | 0.76 | 0.48-1.20 |
| Aphasia | 0.98 | 0.55-1.75 |
| Dysarthria | 1.05 | 0.67-1.66 |
| Aphasia + Dysarthria | 1.85 | 0.88-3.91 |

*reference category men. Adjusted for sex, stroke severity, stroke localization, history of hypertension, diabetes, atrial fibrillation, and hypercholesterolemia. Eye deviation and no symptoms were removed from analysis, due to zero observation in one of the groups.

| **Atypical stroke symptoms** | **OR** | **95 % confidence intervals** |
| --- | --- | --- |
| Headache | 1.18 | 0.71-1.98 |
| Pain | 2.75 | 0.96-7.90 |
| Loss of consciousness | 2.12 | 1.08-4.18 |
| Confusion | 1.20 | 0.68-2.14 |
| Reduced attention | 0.32 | 0.03-3.16 |
| Memory loss | 1.63 | 0.61-4.31 |
| Vertigo | 0.99 | 0.64-1.53 |
| Nausea + vomiting | 2.32 | 1.24-4.35 |
| Dysphagia | 0.82 | 0.36-1.85 |
| Seizure | 3.87 | 0.31-48.53 |
| Sensory changes | 1.21 | 0.76-1.92 |
| Other | 1.76 | 1.05-2.94 |

*reference category men. Adjusted for sex, stroke severity, stroke localization, history of hypertension, diabetes, atrial fibrillation, and hypercholesterolemia. Eye deviation and no symptoms were removed from analysis, due to zero observation in one of the groups.

**Data supplement Table V Age differences in patient-reported stroke symptoms** **(5 years interval)**

| **Typical stroke symptoms (BEFAST)** | **OR** | **95 % confidence intervals** |
| --- | --- | --- |
| Affected balance | 1.03 | 0.91-1.15 |
| Vision disturbance | 0.91 | 0.82-1.00 |
| Facials paresis | 0.98 | 0.89-1.08 |
| Paresis, (arm) | 0.89 | 0.82-0.97 |
| Paresis, (leg) | 1.01 | 0.92-1.10 |
| Ataxia, (arm) | 0.95 | 0.87-1.03 |
| Ataxia, (leg) | 0.98 | 0.89-1.07 |
| Aphasia | 1.06 | 0.94-1.20 |
| Dysarthria | 1.04 | 0.94-1.15 |
| Aphasia + Dysarthria | 1.12 | 0.94-1.33 |

*reference category men. Adjusted for sex, stroke severity, stroke localization, history of hypertension, diabetes, atrial fibrillation, and hypercholesterolemia. Eye deviation and no symptoms were removed from analysis, due to zero observation in one of the groups.

| **Atypical stroke symptoms** | **OR** | **95 % confidence intervals** |
| --- | --- | --- |
| Headache | 0.90 | 0.81-1.00 |
| Pain | 0.97 | 0.79-1.19 |
| Loss of consciousness | 0.98 | 0.85-1.14 |
| Confusion | 1.11 | 0.98-1.26 |
| Reduced attention | 0.88 | 0.58-1.33 |
| Memory loss | 1.08 | 0.87-1.33 |
| Vertigo | 0.97 | 0.89-1.07 |
| Nausea + vomiting | 0.95 | 0.84-1.09 |
| Dysphagia | 1.37 | 1.11-1.68 |
| Seizure | 1.24 | 0.70-2.19 |
| Sensory changes | 0.79 | 0.72-0.87 |
| Other | 0.93 | 0.84-1.03 |

*reference category men. Adjusted for sex, stroke severity, stroke localization, history of hypertension, diabetes, atrial fibrillation, and hypercholesterolemia. Eye deviation and no symptoms were removed from analysis, due to zero observation in one of the groups.

**Data supplement Table VI Patient reported symptoms. Population I61 (n=39)**

| **Variables** | **Women**  N=16  (8.33%) | **Men**  N=23  (8.01%) | **P-value** |
| --- | --- | --- | --- |
| Typical stroke symptom   - Affected balance - Visual disturbance - Facial paresis - Paresis (arm) - Paresis (leg) - Ataxia (arm) - Ataxia (leg) - Aphasia - Dysarthria - Aphasia and dysarthria | 3 (18.75)  3 (18.75)  4 (25.00)  5 (31.25)  5 (31.25)  2 (12.50)  5 (31.25)  1 (6.25)  2 (12.50)  1 (6.25) | 2 (8.70)  4 (17.39)  3 (13.04)  13 (56.52)  11 (47.83)  10 (43.48)  8 (34.78)  3 (13.04)  7 (30.43)  1 (4.35) | 0.63*  1.00*  0.42*  0.12  0.30  0.07*  0.82  0.63*  0.26*  1.00* |
| Atypical stroke symptoms   - Headache - Pain - Loss of consciousness - Confusion - Reduced attention - Vertigo - Nausea + vomiting - Dysphagia - Seizure - Sensory changes - Symptoms that did not fit in the predefined symptom boxes | 3 (18.75)  2 (12.50)  5 (31.25)  4 (25.00)  3 (18.75)  3 (18.75)  5 (31.25)  1 (6.25)  1 (6.25)  4 (25.00)  4 (25.00) | 7 (30.43)  2 (8.70)  1 (4.35)  5 (21.74)  4 (17.39)  8 (24.78)  5 (21.74)  3 (13.04)  1 (4.35)  2 (8.70)  8 (34.78) | 0.48*  1.00*  0.03*  1.00*  1.00*  0.47*  0.71*  0.63*  1.00*  0.20*  0.73* |

*Fisher’ Exact Test. Reference category men. Eye deviation, memory loss, and no symptoms were removed from the analysis, due to zero observation in one of the groups.

**Data supplement Table VII Patient reported symptoms. Population I63 (n = 309)**

| **Variables** | **Women**  N=114  (59.38%) | **Men**  N=195  (67.94%) | **P-value** |
| --- | --- | --- | --- |
| Typical stroke symptom   - Affected balance - Visual disturbance - Facial paresis - Paresis (arm) - Paresis (leg) - Ataxia (arm) - Ataxia (leg) - Aphasia - Dysarthria - Aphasia and dysarthria | 16 (14.04)  19 (16.67)  32 (28.07)  48 (42.11)  37 (32.46)  37 (32.46)  29 (25.44)  13 (11.40)  27 (23.68)  13 (11.40) | 32 (16.41)  42 (21.54)  43 (22.05)  83 (42.56)  80 (41.03)  57 (29.23)  49 (25.13)  17 (8.72)  43 (22.05)  13 (6.67) | 0.58  0.30  0.23  0.94  0.13  0.55  0.95  0.44  0.74  0.15 |
| Atypical stroke symptoms   - Headache - Pain - Loss of consciousness - Confusion - Reduced attention - Memory loss - Vertigo - Nausea + vomiting - Dysphagia - Sensory changes - Symptoms that did not fit in the predefined symptom boxes | 19 (16.67)  5 (4.39)  11 (9.65)  13 (11.40)  1 (0.88)  3 (2.63)  29 (25.44)  20 (17.54)  8 (7.02)  23 (20.18)  18 (15.79) | 25 (12.82)  3 (1.54)  13 (6.67)  17 (8.72)  4 (2.05)  6 (3.08)  52 (26.67)  12 (6.15)  14 (7.18)  45 (23.08)  20 (10.26) | 0.35  0.15*  0.34  0.44  0.66  1.00*  0.81  0.001  0.96  0.55  0.15 |

*Fisher’ Exact Test. Reference category men. Eye deviation, seizure, and no symptoms were removed from the analysis, due to zero observation in one of the groups.

**Data supplement Table VIII Patient reported symptoms. Population G45 (n= 131)**

| **Variables** | **Women**  N=62  (32.29%) | **Men**  N=69  (24.04%) | **P-value** |
| --- | --- | --- | --- |
| Typical stroke symptom   - Affected balance - Visual disturbance - Facial paresis - Paresis (arm) - Paresis (leg) - Ataxia (arm) - Ataxia (leg) - Aphasia - Dysarthria - Aphasia and dysarthria | 2 (3.23)  8 (12.90)  11 (17.74)  17 (27.42)  9 (14.52)  8 (12.90)  4 (6.45)  10 (16.13)  17 (27.42)  4 (6.45) | 10 (14.49)  15 (21.74)  7 (10.14)  16 (23.19)  15 (21.74)  16 (23.19)  12 (17.39)  16 (23.19)  13 (18.84)  2 (2.90) | 0.03*  0.18  0.21  0.58  0.29  0.13  0.07*  0.31  0.24  0.42 |
| Atypical stroke symptoms   - Headache - Pain - Loss of consciousness - Confusion - Memory loss - Vertigo - Nausea + vomiting - Dysphagia - Sensory changes - Symptoms that did not fit in the predefined symptom boxes | 10 (16.13)  3 (4.84)  7 (11.29)  8 (12.90)  4 (6.45)  16 (25.81)  3 (4.84)  2 (3.23)  16 (25.81)  15 (24.19) | 10 (14.49)  1 (1.45)  3 (4.35)  10 (14.49)  3 (4.35)  15 (21.74)  3 (4.35)  1 (1.45)  11 (15.94)  8 (11.59) | 0.79  0.34  0.19*  0.79  0.71  0.58  1.00*  0.60  0.16  0.06 |

*Fisher’ Exact Test Reference category men. Eye deviation, reduced attention, seizure, and no symptoms were removed from the analysis, due to zero observation in one of the groups.
